# Management of Dog-Related Penetrating Laryngotracheal Trauma in a Pediatric Patient

**DOI:** 10.7759/cureus.48470

**Published:** 2023-11-07

**Authors:** Sarah L Debs, Rajanya S Petersson, Stephanie J Wong

**Affiliations:** 1 Otolaryngology - Head and Neck Surgery, Virginia Commonwealth University School of Medicine, Richmond, USA; 2 Otolaryngology, Children's Hospital of Richmond at VCU (Virginia Commonwealth University), Richmond, USA

**Keywords:** multi-disciplinary approach, tracheal perforations, dog bite, pediatric, penetrating laryngotracheal trauma

## Abstract

Dog-bite-related laryngotracheal injuries are rare but can be life-threatening. We present a case of penetrating laryngotracheal trauma in a six-year-old male and the management, considerations, and outcomes. The patient suffered extensive laryngotracheal trauma, including near complete tracheal transection, complete thyroid cartilage fracture, crush injury to the cricoid, and multiple tracheal perforations after a dog attack. We review initial management, subsequent airway interventions, multi-disciplinary approach, and airway outcomes. We present one of the few reports describing extensive dog-related penetrating laryngotracheal trauma in a pediatric patient, with successful airway management.

## Introduction

Dog bites afflict approximately 2.5 million children annually in the United States [1]. The most common sites of dog bites are to the face and head and typically occur in children under 12 years old with 50% of fatal dog bites occurring in children under 10 years old. This is likely due to the proximity of a child’s face and head to the muzzle height of a dog.

Penetrating laryngotracheal injury from dog bites is rare in pediatric patients. Sidell 2011 utilized the National Trauma Data Bank to evaluate 1.9 million cases from 2002-2006, of which 633 were laryngeal trauma events with 69 cases involving pediatric laryngeal injury [2]. Severity can vary widely, with the grading scale for laryngotracheal injuries ranging from I to V; grade I represents minor hematoma or laceration without fracture while grade V represents complete separation [3].

We present one of the few reports describing extensive dog-related penetrating laryngotracheal trauma in a six-year-old male, with successful airway management. We discuss the evaluation, subsequent interventions, and requisite multidisciplinary team approach.

## Case presentation

**Figure 1:**
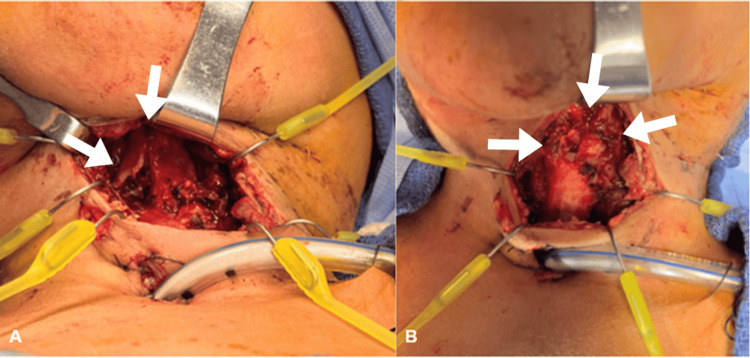
Thyroid cartilage fracture (A) and cricoid crush injury and cricothryoid defect (B) A) White arrows indicate thyroid cartilage fracture; B) White arrows indicate a cricoid crush injury and a cricothyroid defect

A previously healthy six-year-old male was attacked by a Rottweiler resulting in significant scalp and neck injuries. He presented to an outside hospital with a GCS of 8, 12x8 cm full-thickness scalp avulsion with significant tissue loss, and air leaking from his neck wounds. He was intubated at the outside hospital with a 5.5-cuffed endotracheal tube approximately two hours prior to transfer. Subsequent CT revealed a tracheal injury, left vertebral artery dissection, left carotid focal injury, cervical spine fractures, and ligamentous injury. Upon transfer, GCS was 3T with continued air leak through the wounds. The patient was taken urgently to the OR by the Otolaryngology and Pediatric Trauma teams for neck exploration and airway evaluation. The patient's neck had to be kept in a neutral position due to his cervical injuries. There was a large, near-complete, defect in the anterior tracheal wall (Figure 1). There appeared to be minimal cartilage loss with perforation of the 1st and 2nd cartilaginous rings. The ETT through the mouth was replaced with a new 5.0 ETT through the tracheal defect. The distal end of the anterior trachea was sutured to the skin to mature the stoma. Further evaluation by the trauma team noted at least one partial thickness mucosal injury to the esophagus, and a nasogastric (NG) tube was passed and confirmed gastric with an abdominal X-ray. The wounds were copiously irrigated and most of the superficial lacerations were closed. The ETT was then replaced by a 5.5-cuffed Bivona tracheostomy tube. An attempt at flexible and direct laryngoscopy was made, but a view was unable to be obtained due to cervical spine precautions limiting safe neck extension. The pediatric trauma surgery team attempted flexible esophagoscopy but was unable to pass the scope due to the suspected development of edema. An Integra® Bilayer Wound Matrix was cut to fit the scalp defect and secured with xeroform bolster. He was transferred to the PICU in critical but stable condition.

**Figure 2:**
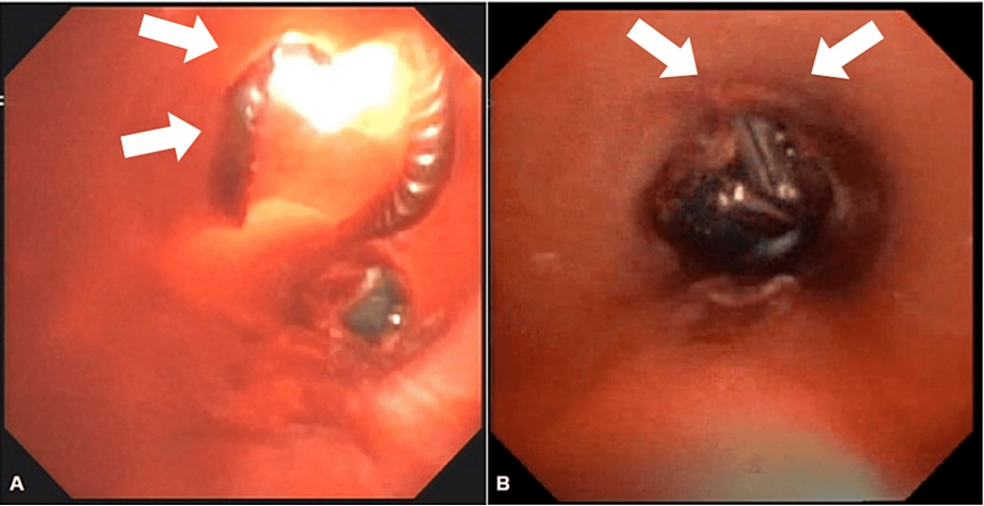
Bronchoscopy with defects visualized Hospital Day 5 (A and B). A) White arrows indicate instrumentation through the thyroid cartilage fracture; B) White arrows indicate instrumentation through the cricothyroid defect

On hospital day 5, the patient underwent neck washout, re-exploration, and laryngotracheal repair with two otolaryngology attendings experienced with pediatric airway reconstruction (Figure 2). Flexible bronchoscopy was significant for hematoma of the right true vocal fold. Neck exploration showed near complete tracheal transection (intact trachealis and sliver of connecting tracheal cartilage but no significant loss of cartilage), complete thyroid cartilage fracture without mucosal violation of the anterior commissure, crush injury to the cricoid, and multiple tracheal perforations. The lateral walls of the tracheal transection, the tracheal perforations, and the thyroid cartilage were reapproximated with 5-0 PDS. Thyroid cartilage was reapproximated with primary repair with two simple interrupted sutures, ensuring submucosal bites. Perforations through the first and second rings were also reapproximated primarily, staying submucosal with the suture. Lateral tracheal shelves were reapproximated primarily with interrupted sutures, and two additional tension sutures were placed around an additional ring superiorly and inferiorly on either side. There was a violation of the cricothyroid membrane; this was covered with strap muscle, which was also reapproximated over the anterior trachea. He was then nasotracheally intubated with a 5.0 ETT to stent the airway, with the cuff confirmed to be inferior to the mid-tracheal injury. A 3.0 Bivona trach was placed alongside the ETT to maintain the stoma. On hospital day 9, the patient underwent flexible bronchoscopy, scalp xeroform bolster replacement, and extubation, after which tracheostomy capping trials were initiated.

On hospital day 20, the patient underwent direct laryngoscopy, bronchoscopy, subglottic balloon dilation with steroid injection, adenoidectomy, and debridement of the scalp. On hospital day 33, he underwent a split-thickness skin graft to the scalp, bronchoscopy, and successful decannulation. Findings included grade 1 (8%) subglottic stenosis and a well-mucosalized trachea. The patient was discharged on hospital day 41 to a pediatric skilled nursing facility.

**Figure 3:**
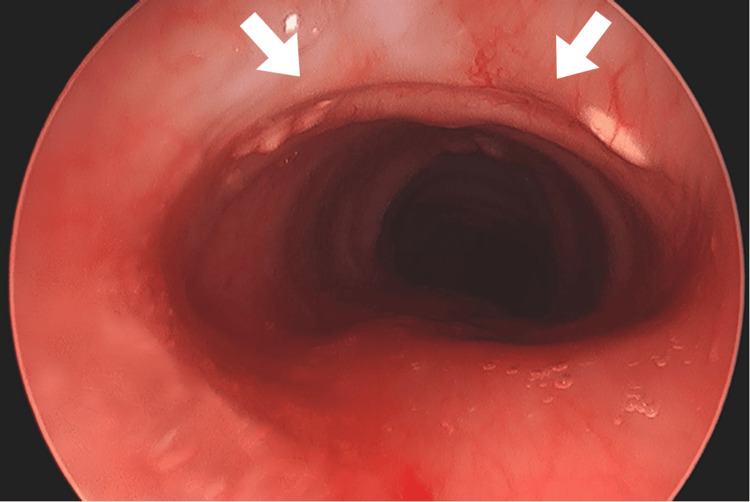
Bronchoscopy with defects visualized 89 days after the initial injury, 56 days after decannulation White arrows indicate well-healed and mucosalized previous sites of a thyroid fracture, cricoid crush injury, and cricothyroid defect

Eighty-nine days after the initial injury, repeat airway evaluation revealed normal-appearing vocal cords and stable subglottic stenosis (Figure 3). His trachea was well-mucosalized and without tracheomalacia or obstruction down to the carina.

## Discussion

Penetrating laryngotracheal trauma is the most common cause of death in patients with head and neck trauma second only to an intracranial hemorrhage [3,4]. Given the potential for near-complete tracheal transection to become complete, extreme care must be taken when securing the airway, especially in a pre-hospital setting. Ideally, intubation would be performed under direct visualization (i.e. flexible bronchoscopy); however, this must be weighed against local resources and acuity of the patient.

Once a stable airway is secured and an understanding of the laryngeal injury is established, the next decision is for observation alone or surgical intervention. Observation is recommended for patients with minor laryngeal edema, small hematomas, or mucosal lacerations without involvement of the anterior commissure [4]. Patients with symptoms such as subcutaneous emphysema, impaired breathing, stridor, or hemoptysis should be considered for surgical intervention as their injuries are less likely to self-resolve and can cause airway compromise [4].

Chang 2019 describes a 10-year-old male who suffered near-complete laryngotracheal separation after blunt cervical trauma sustained due to a bicycle accident [5]. Additional injuries included multiple laryngeal fractures and arytenoid and vocal fold avulsion, which were not noted radiographically. On presentation to the emergency room, the patient was stridulous, and orotracheal intubation was not possible due to a limited view of the airway. The patient underwent an urgent tracheostomy followed by panendoscopy and open neck exploration with the repair of laryngeal fractures and suspension of the avulsed vocal fold. A 9 mm transglottic suprastomal stent was placed from the cricoid to the proximal trachea for one month. The patient was discharged on hospital day 10, decannulated two months after the initial injury, and at the one-year follow-up, noted to be speaking strongly and tolerating a normal diet.

The pediatric patient we described and that in Chang 2019 both sustained near-complete laryngotracheal separations due to trauma. Similarly, they required a definitive airway followed by open neck exploration with surgical repair of laryngeal injuries. Both of the patients had intraoperative transglottic suprastomal stenting and were later able to be decannulated. Additionally, both patients had routine airway evaluations and were noted to be well-healed with functional speech and swallowing.

Garvey 2015 reported a case of a five-year-old male with laryngotracheal transection following a Pit Bull attack [6]. The patient underwent airway evaluation, tracheostomy, and laryngotracheal transection repair. The patient’s care involved a multidisciplinary team and the course was complicated by vocal cord paralysis, laryngotracheal stenosis, and dysphagia ultimately requiring a gastrostomy tube. The patient was hospitalized for a total of 25 days.

We report the second known case in the literature of a pediatric patient who suffered extensive laryngotracheal trauma due to a dog bite. This six-year-old patient had near complete tracheal transection, complete thyroid cartilage fracture, crush injury to the cricoid, and multiple tracheal perforations. This was managed initially by securing the airway, and followed by laryngotracheal repair, tracheal dilation, and routine airway evaluations. Techniques described include temporarily maturing the stoma, the use of ETT as a stent, and maintaining the tracheostomy tube stoma until ready to decannulate. Coordination of care between pediatric trauma, anesthesia, facial plastics, pediatric otolaryngology, respiratory and speech therapy, and ICU was critical [5,6]. The patient was successfully decannulated approximately one month after his injury, and the laryngotracheal framework was well-healed at three months with the patient tolerating all nutrition by mouth.

## Conclusions

Penetrating laryngotracheal trauma is variable in presentation; however, in rare instances, it can progress rapidly to life-threatening airway compromise. Airway management is essential and includes establishing a definitive airway and skilled multidisciplinary care to be successfully decannulated and have good long-term outcomes.
